# Revisiting T Cell Tolerance as a Checkpoint Target for Cancer Immunotherapy

**DOI:** 10.3389/fimmu.2020.589641

**Published:** 2020-09-23

**Authors:** Simone Nüssing, Joseph A. Trapani, Ian A. Parish

**Affiliations:** ^1^Peter MacCallum Cancer Centre, Melbourne, VIC, Australia; ^2^Sir Peter MacCallum Department of Oncology, University of Melbourne, Parkville, VIC, Australia

**Keywords:** cancer immunotherapies, immunological tolerance, cancer immune evasion, CD8^+^ T cell, checkpoint blockade

## Abstract

Immunotherapy has revolutionized the treatment of cancer. Nevertheless, the majority of patients do not respond to therapy, meaning a deeper understanding of tumor immune evasion strategies is required to boost treatment efficacy. The vast majority of immunotherapy studies have focused on how treatment reinvigorates exhausted CD8^+^ T cells within the tumor. In contrast, how therapies influence regulatory processes within the draining lymph node is less well studied. In particular, relatively little has been done to examine how tumors may exploit peripheral CD8^+^ T cell tolerance, an under-studied immune checkpoint that under normal circumstances prevents detrimental autoimmune disease by blocking the initiation of T cell responses. Here we review the therapeutic potential of blocking peripheral CD8^+^ T cell tolerance for the treatment of cancer. We first comprehensively review what has been learnt about the regulation of CD8^+^ T cell peripheral tolerance from the non-tumor models in which peripheral tolerance was first defined. We next consider how the tolerant state differs from other states of negative regulation, such as T cell exhaustion and senescence. Finally, we describe how tumors hijack the peripheral tolerance immune checkpoint to prevent anti-tumor immune responses, and argue that disruption of peripheral tolerance may contribute to both the anti-cancer efficacy and autoimmune side-effects of immunotherapy. Overall, we propose that a deeper understanding of peripheral tolerance will ultimately enable the development of more targeted and refined cancer immunotherapy approaches.

## Introduction

In the last decade, immunotherapy has revolutionized the treatment of cancer. In particular, immune checkpoint blockade by individual or combined treatment with FDA approved drugs, such as α-PD1/α-PD-L1 (nivolumab, pembrolizumab), α-CTLA-4 (ipilimumab), and α-Lag3 [relatlimab, in clinical trials (1)] antibodies (2), has shown considerable promise in the treatment of multiple cancers, including melanoma, non-small-cell lung cancer, head and neck squamous cell carcinoma, Hodgkin lymphoma, and renal cell carcinoma (3, 4). Nevertheless, in most cancer types the majority of patients do not respond to therapy, while other cancers are largely therapy resistant. For this reason, there is considerable interest in both identifying approaches that can amplify therapeutic efficacy, in addition to better delineating the fundamental biology underpinning the response to therapy.

CD8^+^ T cells are considered one of the main targets of cancer immunotherapy (5). In particular, there is strong evidence that a primary mechanism underlying treatment success is reinvigoration of tumor infiltrating CD8^+^ T cells whose function has been diminished via a process called exhaustion. Tumor infiltration by exhausted CD8^+^ T cells is a strong predictor of response to therapy (6, 7) and pre-clinical animal studies have indicated that certain exhausted cell subsets are required for therapeutic efficacy (5, 8–10). For this reason, the vast majority of recent work in the field has focused on the factors that regulate T cell infiltration and function within tumors. However, recent data indicate that T cell responses initiated outside the tumor are major contributors to anti-tumor efficacy during immunotherapy (11–13). Thus, a better understanding of how anti-tumor responses are initiated, and how tumors evade this process, is critical to the development of more efficacious immunotherapy approaches.

In this review, we argue that induction of CD8^+^ T cell peripheral tolerance within the draining lymph node is a major contributor to tumor immune evasion, and that disruption of this immune checkpoint may contribute to the efficacy of cancer immunotherapy. We first summarize what is known about CD8^+^ T cell peripheral tolerance from work done in autoimmune models, and contrast peripheral tolerance with other states of negative regulation, such as exhaustion and senescence. We then review the evidence that tumors hijack peripheral tolerance to evade anti-cancer immunity, and that tolerance disruption by immunotherapy both augments anti-cancer efficacy and contributes to autoimmune adverse events. Overall, we argue that a more detailed understanding of peripheral tolerance to tumors will aid the development of more refined therapies that maximize anti-tumor immunity while limiting deleterious side-effects.

## T Cell Tolerance: What Is It and Why Does It Matter?

The random recombination events that generate T cell receptors (TCRs) during thymic development will inevitably produce TCR specificities that could be damaging to the host. In particular, self-reactive TCRs will be generated, in addition to TCRs specific for harmless environmental antigens, such as food-derived antigens and pollens. Failure to refine the TCR repertoire to limit these damaging specificities is detrimental to the host. For this reason, a suite of mechanisms collectively referred to as tolerance mechanisms have evolved to either eliminate or restrain these potentially dangerous TCR specificities. The collective importance of tolerance mechanisms to host health is best illustrated by the consequences of genetic deficiencies in the tolerance process. For example, deficiencies in genes critically required for T cell tolerance, such as *AIRE* and *FOXP3*, can lead to devastating autoimmune disease in both patients and mice (14–19).

The vast majority of self-reactive T cells are purged from the repertoire during thymic development. Recognition of self-antigens present in the thymus during development triggers death by negative selection (20), and given that processes exist to enable ectopic expression of tissue-restricted antigens within the thymus (21), this process is capable of eliminating a large proportion of the self-reactive cells that are generated during T cell development. However, this process is not perfect. First, as self-reactivity can occur across a range of TCR-self-antigen affinities, and not only when a clearly defined threshold is exceeded, by necessity some degree of self-reactivity must be tolerated by the host. Consistent with this idea, previous studies have demonstrated that 25–40% of antigen-specific T cells escape thymic selection to a ubiquitously expressed antigen, with these “escapees” typically enriched for lower affinity TCRs (22). Furthermore, not every peripheral self-antigen is likely to be expressed in the thymus, and even when expression does occur, there is no guarantee that the antigen will be appropriately processed and presented to effect selection (23). Consistent with these ideas, self-reactive T cells can be found in the periphery of otherwise healthy hosts (24–28). In fact, in a recent study, T cells reactive for the ubiquitous male H-Y antigen were found in the periphery of men at similar precursor frequencies to that of pathogen-specific T cells (29). Leakiness in thymic selection likely exists because, despite increasing the risk of autoimmunity, it broadens the T cell repertoire against pathogens and thereby prevents pathogens from exploiting selection-induced “holes” in the repertoire (29). For example, certain pancreatic islet-reactive CD8^+^ T cell specificities are cross-reactive to a commensal bacterium in the gut (26). Nevertheless, the stream of self-reactive cells that escapes into the periphery after thymic selection can also pose a risk, and for this reason a range of tolerance mechanisms have evolved in the periphery to quell these potentially dangerous cells.

## The Peripheral T Cell Tolerance Immune Checkpoint

Broadly speaking, peripheral T cell tolerance refers to the range of mechanisms that can prevent or limit naïve T cell activation in the periphery. While peripheral tolerance is likely to predominantly function to restrain self-reactive T cells, it ultimately can limit activation of any TCR specificity that is engaged by antigen in conditions that favor tolerance induction. These peripheral tolerance mechanisms can lead to a range of outcomes, including a failure to become activated at all (ignorance), persistence of responding cells in a refractory or “anergic” state, and T cell elimination by apoptotic death (“deletion”) (30–32). Typically, peripheral tolerance occurs only in the absence of infection and inflammation. Anergy or deletion are the “default” programs triggered when a naïve T cell encounters its antigen within secondary lymphoid organs in the absence of innate immune activation by infection-associated signals (e.g., pathogen-derived products or pathological tissue damage) (32, 33). The common feature of all peripheral tolerance mechanisms is that the responding T cells typically fail to develop effector functions, with cells also often failing to clonally expand or infiltrate peripheral tissues. Thus, the ultimate result of all peripheral tolerance mechanisms is that the target T cells are prevented from participating in an immune response. Tolerising cells that respond in the absence of infection or inflammation is an efficient way of selectively quelling self-reactive cells, as cells responding to antigen in the absence of infection/inflammation are likely responding to self-antigens, and thus are dangerous to the host. Alternatively, cells may be responding to harmless environmental antigens, in which case tolerising these cells is also beneficial to prevent unnecessary inflammatory responses. However, as will be discussed later, certain tumors hijack peripheral tolerance processes to limit anti-tumor immunity. A range of cell extrinsic and intrinsic factors guide both whether or not tolerance occurs, and the type of tolerance mechanism induced. Most of these factors have been elucidated in autoimmune models, but it is important to review these findings, as the same principles almost certainly hold true in cancer.

## Anergy, Deletion, or Ignorance: It Is All About Antigen Access and Dose

Studying peripheral tolerance induction within the self-reactive T cells of unmanipulated mice is inherently difficult, as the main feature of tolerance is absence of a T cell response. For this reason, much of what we have learned about *in vivo* CD8^+^ T cell peripheral tolerance has been deduced from transgenic mouse models. In these systems, a model antigen, such as chicken ovalbumin (OVA), allo-MHC or a viral antigen, is transgenically expressed as a “neo-self antigen” under a tissue-specific promoter. Adoptive transfer of antigen-specific TCR transgenic cells, or infection with a pathogen to induce a response against the model antigen that can be measured by MHC tetramers, is then used to define the rules governing tolerance to the model antigen. A range of such models have been generated that express model antigens within diverse organs, including the pancreas (34–37), gut (38), skin (39, 40), brain (41), parenchymal cells (42), haematopoietic cells, (43), and CD11c^+^ cells (44). While these models have proven useful in defining the rules underpinning peripheral T cell tolerance, they have obvious caveats. First, it is unclear how representative high affinity TCR transgenic cells are of the endogenous self-reactive cells normally subjected to peripheral tolerance. Second, these models utilize viral or foreign antigens as model self-proteins, and these antigens may not be representative of the self-antigens that normally drive peripheral tolerance. Notably, the self epitopes recognized during autoimmune disease are often atypical in their features and MHC binding characteristics (45). Finally, in these models, TCR transgenic cells are often studied at numbers that are many orders of magnitude greater than the physiological frequency of antigen-specific T cells, which may influence outcome (see later discussion on precursor frequency in tumor models). Nevertheless, from this broad range of model systems, which employ a range of model antigens, TCR specificities and affinities, and target organs, a number of common peripheral tolerance principles have emerged.

Some of the first transgenic peripheral tolerance models established the concept of ignorance. In these systems, it was possible to trigger an autoimmune effector CD8^+^ T cell response against the model transgenic antigen by either IL-2 over-expression (46) or viral infection (47, 48). It could therefore be concluded that a repertoire of naïve “self-antigen”-specific T cells normally exists in these hosts, meaning that there is no peripheral process for eliminating the self-reactive cells that escape thymic selection in these models. This phenomenon was termed T cell “ignorance” (30), and led to the idea that self-tolerance is maintained in the periphery because self-antigens are sequestered within tissues, and thus self-reactive T cells are never engaged. However, subsequent studies challenged this idea, as in numerous other transgenic models, constitutive self-antigen presentation was observed in the periphery, and such steady-state antigen recognition typically led to either T cell deletion or anergy (34–36, 38–40, 42–44, 49–54). A seminal study subsequently explained the discrepancy between these findings by linking tolerance outcome to model antigen expression levels (34). In two distinct transgenic mouse strains expressing the same model antigen (OVA) at different levels within the pancreas, low OVA expression led to ignorance due to insufficient antigen presentation in the draining lymph node, while higher OVA levels precipitated a response that ultimately caused peripheral deletion. Additionally, certain tissues, such as brain (41), may be associated with ignorance due to greater efficiency at sequestering antigen from immune recognition. Thus antigen access, which is often linked to antigen expression level, dictates whether or not ignorance occurs.

When a self-antigen is encountered in the absence of infection or appropriate inflammatory signals, the responding T cells typically fail to acquire effector functions (cytokine production, cytotoxic capacity) regardless of whether the cells are deleted or persist in an anergic state (44, 55–57). CD8^+^ T cells will occasionally transiently pass through an effector phase *en route* to tolerance (58, 59), or gain restricted function (e.g., cytotoxicity but not cytokine function) (38), but tolerised cells are invariably functionally impaired relative to conventional effector T cells. Whether or not the tolerant cells persist in an anergic state or are deleted is again linked to antigen load. High antigen loads typically lead to anergy, while lower antigen levels trigger deletion (60, 61). This concept explains why some tolerance models induce anergy while others trigger deletion; anergy is invariably seen in transgenic models where the target model self-antigen is systemically expressed at high levels [e.g., (44)], while deletion typically occurs in models with low level, tissue-specific antigen expression [e.g., (35, 54)]. The molecular mechanisms enabling T cell persistence in anergy models are unclear, however, we speculate that the high antigen loads in these models keep T cells alive that would otherwise die, and that anergy and deletion are otherwise very similar states. In support of this idea, the transcriptional profile of CD8^+^ T cells undergoing deletion strongly overlaps with that seen in anergy (56), and blocking cell death in some deletion models results in anergy (62). Nevertheless, we recently found that anergy and deletion have distinct molecular dependencies (63) (see discussion on NDFIP1 below), meaning that there may be differences between these states. Overall, though, the functional outcome of both anergy and deletion is the same: inactivation of the responding T cell clones to prevent them from participating in future immune responses. A range of cell extrinsic factors, in addition to certain intracellular pathways, control the tolerised state, and these are reviewed in the following sections.

## Cell Extrinsic Factors That Regulate Peripheral Tolerance

**FIGURE 1:**
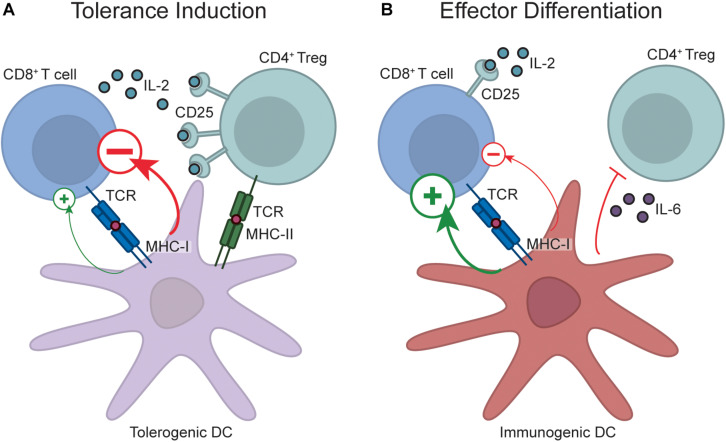
Regulation of CD8^+^ T cell peripheral tolerance by DCs and Tregs. During tolerance induction **(A)**, naïve CD8^+^ T cells encounter antigen on resting, “tolerogenic” DCs. These DCs transmit a net negative signal to responding T cells, which prevents effector differentiation and diverts T cells down a fate of tolerance. Tolerance induction also requires Treg engagement with the DC, and Treg-DC interactions enable proximity-based IL-2 quenching via CD25, and also potentially enforce tolerance by Treg-mediated DC conditioning. In contrast, during effector differentiation **(B)**, DCs transition into an immunogenic state upon recognition of pathogen-derived products and pathological tissue damage. Antigen recognition on immunogenic DCs programs acquisition of an effector fate due to delivery of a net positive signal. Additionally, inflammatory cytokines produced by immunogenic DCs, such as IL-6, may inhibit Treg-mediated immunosuppression by rendering CD8^+^ T cells resistant to Treg suppression, while production of other inflammatory cytokines during infection or sterile inflammation, such as IL-12, may also act directly on CD8^+^ T cells to promote effector differentiation. Note that despite the role of factors like PD-L1 in tolerance induction, it is not possible to distinguish immunogenic from tolerogenic DCs by the expression of any single marker, and instead the overall balance of positive and negative signals likely dictates outcome.

Peripheral tolerance processes such as deletion and anergy occur when naïve T cells encounter their cognate antigen within a secondary lymphoid organ in the absence of infection or inflammatory signals. As T cells recognize their antigen in the context of MHC molecules, encounters between antigen presenting cells and T cells are a necessary initiating event in the tolerance process. Dendritic cells (DCs) have emerged as key players in this initial antigen recognition event and, in the case of CD8^+^ T cell peripheral tolerance, the cDC1 subset of DCs is the key initiator of this process. cDC1 cells are uniquely endowed with the capacity to “cross-present” exogenous antigen on MHC Class I (64), and these cells constitutively capture and cross-present self-antigens to CD8^+^ T cells, which is a critical component of tolerance induction (36, 65, 66). The importance of this process was illustrated by the finding that endogenous self-reactive CD8^+^ T cells accumulate in mice with a deficiency in cross-presentation (67). However, cDC1 cells are also critically required for induction of effector CD8^+^ T cell responses (68, 69), which raises the question of how cDC1s can both activate and inhibit CD8^+^ T cell responses. The explanation is that DCs are plastic in phenotype; in the absence of infection or tissue damage, antigen is presented in a context that induces tolerance, however, upon sensing pathogen derived products and/or pathological tissue damage, or receiving “help” from effector CD4^+^ T cells, DCs transition into an immunogenic state and provide signals to facilitate effector T cell differentiation (68–70) (Figure 1). The identity of the specific DC-derived signals that enable CD8^+^ T cell tolerance versus immunity remain unclear. Initial work in *in vitro* anergy models suggested that anergy occurs when T cells encounter antigen in the absence of co-stimulation (71), leading to the idea that limited co-stimulatory molecule expression on resting DCs was the mechanism by which tolerance induction occurred (70). However, a number of studies have demonstrated that DCs are capable of inducing tolerance even when they express abundant surface co-stimulatory molecules, which has challenged this hypothesis (70). Recent transcriptomic studies have further indicated that there is no single marker that explains the differential function of “tolerogenic” versus “immunogenic” DCs (72), although certain broad signaling pathways such as IFN-I signaling (72) and the NF-κB1 pathway (73) are associated with DC immunogenicity. Instead, the outcome of a T cell-DC interaction is likely dictated by the integrated sum of negative and positive signals encountered, consistent with recent studies demonstrating that T cells sum different inputs during differentiation (74). Nevertheless, shifting this balance by ablation of certain inhibitory pathways is sufficient to breach tolerance. For example, loss of the PD-1 and/or CTLA-4 signaling pathways is sufficient to disrupt peripheral CD8^+^ T cell tolerance (75–80), although the effects of CTLA-4 blockade may be due to indirect effects on regulatory T cells (see below). Despite the critical importance of these pathways, expression levels of ligands for these pathways (such as PD-L1) do not identify “tolerogenic” DCs (72), again arguing that the overall balance of signals delivered by the DC to the T cell is the defining factor governing whether or not tolerance occurs. These DC-derived signals likely synergise with elevated inflammatory cytokines during infection or inflammation to promote effector CD8^+^ T cell differentiation. Indeed, although insufficient to fully breach tolerance, IL-12 can partially rescue CD8^+^ T cell function in tolerance models (59).

Foxp3^+^ regulatory T cells (Tregs) can also influence peripheral T cell tolerance. The evidence often cited to support this idea is that acute or chronic Treg depletion leads to severe inflammatory and autoimmune disease (15, 81). However, the diverse effects that Tregs can have on the immune response (82), together with their effects on tissue homeostasis (83), makes it challenging to determine whether autoimmunity observed upon Treg depletion is due to disrupted peripheral tolerance. For example, it is possible that the inflammatory phenotypes seen upon Treg depletion result because Tregs normally restrain self-reactive effector T cells that may have escaped peripheral tolerance, and these pre-existing effector cells are “unleashed” upon Treg depletion. However, strong evidence exists that constitutive “quenching” of IL-2 by Tregs is an important and direct contributor to peripheral tolerance induction. IL-2 treatment is sufficient to breach *in vivo* CD8^+^ T cell peripheral tolerance (84), and the IL-2 liberated by Treg depletion disrupts *in vivo* CD8^+^ T cell peripheral tolerance (85). Moreover, sophisticated experiments utilizing mice with Tregs incapable of IL-2 quenching led to accumulation of activated CD8^+^ T cells (86), although this could again be due to effects on existing self-reactive effector cells rather than impaired peripheral tolerance induction. A caveat of these experiments is that the phenotypes caused by IL-2 liberation upon Treg depletion may not be representative of the normal, steady-state function of Tregs. For example, excessive IL-15 signaling can also breach CD8^+^ T cell peripheral tolerance (87, 88), but IL-15-consuming memory T cells would not generally be regarded as regulatory. However, recent compelling evidence has identified constitutive IL-2 quenching by Tregs. Imaging studies demonstrated TCR-dependent localization of IL-2 sensing Tregs alongside putative self-reactive, IL-2 producing T cells within unmanipulated wild-type mice (89).

Another mechanism by which Tregs may contribute to peripheral tolerance induction is through conditioning of DCs. A recent compelling study found that conditional MHC Class II ablation from cross-presenting cDC1s disrupted CD8^+^ T cell peripheral tolerance, presumably due to impaired interactions between Tregs and cDC1s (90). These effects are likely due to the large range of direct effects that Tregs have on DC immunogenicity (82), in addition to enabling Treg recruitment to adjacent self-reactive CD8^+^ T cells for proximity-dependent IL-2 quenching. Thus, strong data suggest that Treg dependent IL-2 quenching and/or DC conditioning play non-redundant roles in enforcing CD8^+^ T cell tolerance (Figure 1). Given that CTLA-4 blockade largely boosts immunity through Treg depletion (91), CTLA-4 targeting antibodies previously reported to disrupt peripheral CD8^+^ T cell tolerance (76, 77) may primarily function by Treg depletion rather than direct effects on CD8^+^ T cell-intrinsic CTLA-4 function. This has relevance when considering how CTLA-4 targeting for cancer immunotherapy may influence peripheral tolerance induction.

As Tregs are constitutively present in the host, an obvious question is how effector CD8^+^ T cells are able to evade Treg suppression during the initiation of a response to an infection. While this question has not been definitively answered for CD8^+^ T cells, data from CD4^+^ T cells suggest that inflammatory cytokines induced by pathogen-derived products, such as IL-6, may render effector cells resistant to Treg-mediated suppression thereby enabling effector differentiation (92, 93). To what degree similar pathways operate during effector CD8^+^ T cell differentiation is currently unclear. It is also possible that IL-2 induction by the positive signals delivered by immunogenic DCs, combined with IL-2 from activated CD4^+^ T cells, elevates IL-2 to a level that overwhelms the regulatory T cell pool.

## Transcriptional and Signaling Pathways That Enforce Peripheral Tolerance

Surprisingly little is understood about the T cell-intrinsic molecular pathways that enforce the tolerant state of CD8^+^ T cells. Instead, most of our knowledge on the pathways that enforce peripheral tolerance comes from studies of *in vitro* CD4^+^ T cell anergy. While most of these pathways have been validated within *in vivo* CD4^+^ T cell tolerance models, it is unclear to what extent they operate during CD8^+^ T cell tolerance. Nevertheless, circumstantial, and in some cases direct, evidence suggests that at least some of these pathways operate similarly in both cell types.

**FIGURE 2:**
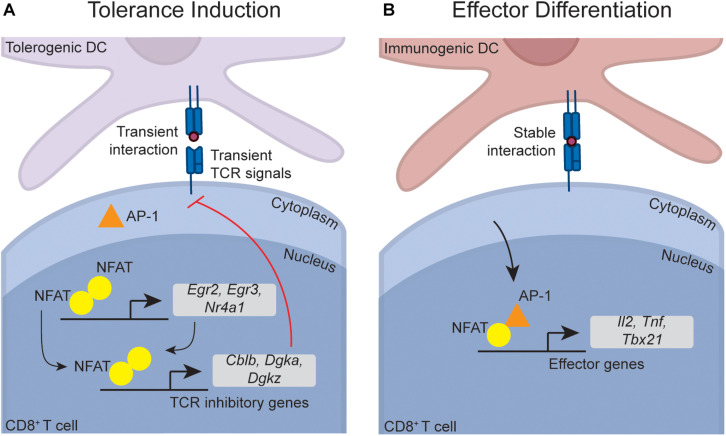
Putative transcriptional pathways that program CD8^+^ T cell tolerance. Although not directly demonstrated within CD8^+^ T cells, data suggest that the NFAT-dependent transcriptional programs that induce CD4^+^ T cell anergy also operate during CD8^+^ T cell tolerance. During tolerance induction **(A)**, transient naïve CD8^+^ T cell interactions with DCs (which can occur due to PD-1 and CTLA-4 signaling) lead to an accumulation of NFAT in the nucleus in the absence of AP-1. This is because AP-1 is rapidly exported from the nucleus upon antigen withdrawal, while NFAT is slowly exported, meaning that transient interactions lead to preferential nuclear NFAT accumulation. In the absence of AP-1, NFAT forms low affinity homodimers that induce transcription factors such as *Egr2*, *Egr3*, and *Nr4a1* (also called Nur77). These transcription factors, in combination with NFAT, induce a range of factors that directly inhibit TCR signaling (such as *Cblb*, *Dgka*, and *Dgkz*), thereby enforcing anergy. During effector differentiation **(B)**, stable DC-T cell interactions enable sustained TCR signaling, leading to accumulation of both AP-1 and NFAT within the nucleus. NFAT-AP-1 complexes are higher affinity than NFAT homodimers, meaning that in the presence of AP-1, NFAT-AP-1 complexes preferentially form. When complexed with AP-1, NFAT preferentially induces effector genes such as the cytokines *Il2* and *Tnf*, and *Tbx21* (encoding TBET), which in turn induces *Ifng*.

NFAT activation and nuclear translocation downstream of TCR-induced calcium signaling is a central pathway required for CD4^+^ T cell anergy (94). However, NFAT also plays indispensable roles in T cell activation (95), suggesting that NFAT function must be modulated in anergy versus effector differentiation. Seminal studies within CD4^+^ T cells have shown that whether or not the transcription factor AP-1 is activated during initial TCR signaling defines whether or not NFAT induces a tolerant or effector gene program (Figure 2). NFAT activation in the absence of AP-1 activation and nuclear translocation leads to the formation of low affinity NFAT homodimers that target inhibitory anergy-associated genes, while simultaneous AP-1 and NFAT activation triggers preferential formation of a higher affinity NFAT-AP-1 complex that targets effector genes, such as genes for the cytokines IL-2 and TNFα and the transcription factor TBET (which in turn can induce the cytokine IFNγ) (94, 96–101). Importantly, these signaling pathways operate similarly within effector CD8^+^ T cells (98), and a range of NFAT-dependent anergy gene targets are selectively induced within tolerant CD8^+^ T cells *in vivo* (e.g., *Egr2*, *Nr4a1*, *Cblb*, *Dgkz*) (56), providing circumstantial proof that these pathways operate similarly during CD8^+^ T cell tolerance. It was unclear, though, how TCR engagement during tolerance induction would selectively trigger activation of NFAT but not AP-1, as restimulation of naïve T cells with peptide alone on *in vitro* splenocytes from an uninfected mouse induces activation of both signaling pathways [for example, (56)]. However, an elegant intravital imaging study provided a solution to this paradox by demonstrating that TCR signaling is influenced by the unique nature of T cell-DC interactions during tolerance induction. Unlike effector differentiation, where T cells formed prolonged contacts with immunogenic DCs, T cells fail to form prolonged contacts with tolerogenic DCs *in vivo*, and this directly contributes to limited effector differentiation during tolerance (102, 103). Intravital imaging has demonstrated that NFAT is more slowly exported from the T cell nucleus than AP-1 after antigen withdrawal *in vivo*, and thus CD8^+^ T cells that experience transient DC interactions have increased periods of time with NFAT unaccompanied by AP-1 within the nucleus. This leads to preferential induction of anergy rather than effector program-associated genes (104). Overall, these data suggest that transient interactions between CD8^+^ T cells and DCs during peripheral tolerance induction are responsible for inducing NFAT-dependent regulatory programs. Interestingly, both PD-1 and CTLA-4 promote transient rather than stable T cell-DC interactions (105, 106), providing an additional mechanism by which these immune checkpoints may contribute to tolerance induction. Nevertheless, definitive functional genetic knock-out data specifically demonstrating that the NFAT-dependent anergy program enforces *in vivo* peripheral CD8^+^ T cell tolerance is lacking. Interestingly, a similar paradigm has been proposed in CD8^+^ T cell exhaustion, where weak NFAT homodimers also contribute to induction of the exhaustion gene program (98). This raises interesting questions about whether similar regulatory gene programs are employed in both exhaustion and anergy, a topic that will be discussed in detail later in this review.

NFAT enforces tolerance within CD4^+^ T cells in part through induction of the transcription factors EGR2, EGR3, and NR4A1, which in turn induce downstream genes that inhibit function (107–110). These three transcription factors also restrain the function of established effector CD8^+^ T cells (110–112), and are induced within tolerant CD8^+^ T cells (56), although it is again unclear whether they functionally contribute to *in vivo* CD8^+^ T cell peripheral tolerance. As discussed later in this review, various lines of evidence also implicate these transcription factors in the control of exhaustion, which is again an interesting parallel between both tolerance and exhaustion. Independent of the NFAT-AP-1 paradigm, c-REL has also been implicated as a factor that inhibits CD8^+^ T cell anergy and promotes effector differentiation. c-REL deficient CD8^+^ T cells aberrantly become anergic *in vitro* under conditions that would normally favor effector differentiation, although cells unable to activate c-REL differentiated normally during infection *in vivo*, indicating that other factors can compensate for c-REL deficiency (113, 114). It is possible that c-REL cooperates with AP-1 in redirecting NFAT function towards effector genes, although this idea has not been tested.

The hallmark feature of anergic T cells is impaired TCR signaling, which prevents anergic cells from responding to a subsequent challenge (115, 116). NFAT plays an important role in restraining anergic CD4^+^ T cell TCR signaling, as the majority of the downstream genes induced by NFAT (either directly, or indirectly via EGR2, EGR3, and NR4A1) are factors that limit TCR signaling (Figure 2). This includes a number of E3 ubiquitin ligases (such as CBL-B, ITCH, and GRAIL) that degrade TCR signaling components (107–109, 117), phosphatases that block key signaling events (e.g., DGKα and DGKζ) (107–109, 118, 119), and inhibitory receptors that proximally inhibit TCR signaling (e.g., PD-1, TIM3, and LAG3) (98). These factors collectively inhibit TCR-induced ERK and mTOR signaling within anergic CD4^+^ T cells (71). Similar signaling deficiencies are evident in *in vivo* CD8^+^ T cell peptide anergy models (61, 63), and CD8^+^ T cells undergoing peripheral tolerance up-regulate many of these NFAT-dependent inhibitory genes (56). This suggests that active inhibition of TCR signaling also enforces CD8^+^ T cell tolerance. Consistent with this idea, we recently found that *in vivo* CD8^+^ T cell anergy is dependent upon the adaptor protein NDFIP1, which is known to enforce *in vitro* CD4^+^ T cell anergy by promoting the function of a range of anergy-associated ubiquitin ligases (63). NDFIP1 likely limits anergic CD8^+^ T cell function at least in part through inhibition of TCR signaling (63), consistent with the idea that inhibition of TCR signaling also contributes to CD8^+^ T cell anergy. However, in contrast to anergy, NDFIP1 was dispensable for CD8^+^ T cell deletion (63), and we have also found that CBL-B deletion has little impact upon peripheral CD8^+^ T cell deletion (I.A. Parish, unpublished observations) despite evidence that CBL-B is required for peptide-induced CD8^+^ T cell anergy (120). Thus, inhibition of TCR signaling is not required for peripheral deletion of CD8^+^ T cells. Consistent with this idea, TCR signaling defects are less evident in CD8^+^ T cell deletion versus anergy (56, 61). Nevertheless, circumstantial data suggest that activation of the mTOR signaling pathway can disrupt peripheral CD8^+^ T cell deletion. As noted earlier, both IL-2 treatment, and PD-1 and CTLA-4 blockade, can disrupt peripheral deletion (75, 77, 84), and both of these perturbations strongly activate the mTOR signaling pathway (121, 122). Overall, though, functional data examining the role of TCR signal inhibition in the *in vivo* CD8^+^ T cell peripheral tolerance process is still limited and further work is needed.

In contrast to other aspects of the CD8^+^ T cell peripheral tolerance process, the pathways responsible for apoptotic cell death during peripheral deletion are relatively well characterized. Cell death is regulated through the mitochondrial pathway by cell intrinsic BCL2 rather than through death receptors, with apoptotic death specifically dependent on the pro-apoptotic BH3-only protein BIM (123, 124). This is linked to transcriptional upregulation of *Bim* expression during deletion (56), although post-transcriptional regulation of BIM activity has not been excluded as a possible additional regulatory mechanism. While BIM also controls cell death during contraction of an effector CD8^+^ T cell response, there are some distinctions between the *Bim* induction pathways in tolerant versus effector cells. FOXO3 is required for *Bim* induction and cell death during contraction of effector cells (125, 126), but dispensable for *Bim* induction and death of tolerant cells (127), suggesting that distinct regulatory pathways control cell death in tolerance versus effector differentiation. The transcriptional regulation of *Bim* induction during tolerance remains undefined, but may be EGR2-dependent given that *Egr2* is induced during CD8^+^ T cell deletion (56), and *Bim* is a direct EGR2 target in anergic CD4^+^ T cells (128).

It is unclear whether the differentiation state induced by CD8^+^ T cell peripheral tolerance is epigenetically stable, or indeed, whether peripheral tolerance even induces a distinct epigenetic state. Conflicting data exist on this point. Removal of tolerant cells from a peripheral deletion model and transfer into antigen-free hosts followed by a 4 week “rest” period enabled both survival, and subsequent effector differentiation, of the transferred cells after rechallenge with a viral infection (129). Thus, persistent antigen is required to program tolerance in this model, and differentiation competency is recovered in cells “rested” away from antigen. This is similar to findings in *in vivo* CD4^+^ T cell anergy models, where cells recovered TCR signaling capacity after withdrawal from antigen (130). In contrast, in certain contexts CD8^+^ T cell tolerance may be epigenetically stable. Isolation of anergic CD8^+^ T cells and transfer into lymphopenic hosts enabled proliferative expansion, however, when the cells were placed back into a lymphoreplete host they retained a “memory” of their anergic state (131). A caveat of this study was that the tolerant population had encountered their antigen in the thymus, making it unclear to what degree programming in the periphery had induced epigenetic commitment to tolerance. However, similar results have been seen in an independent adoptive transfer-based CD8^+^ T cell anergy model (76) suggesting that epigenetic commitment can occur in multiple tolerance models. These conflicting results are likely related to the timeframe of analysis. In the study where no epigenetic programming was observed, cells were isolated 4 days after tolerance induction, whereas the latter studies observed commitment to tolerance after more prolonged exposure to antigen. Given that cells do not persist in deletion models, the implication is that stable epigenetic programming is unlikely to occur during peripheral deletion, as cells likely die before epigenetic commitment occurs. Nevertheless, more work is needed to clarify the nature and timeframe of epigenetic commitment to the tolerant state.

## Where Does CD8^+^ T Cell Tolerance Fit in the Differentiation Hierarchy?

In the past 15 years, great strides have been made in our understanding of the dynamics of effector, memory and exhausted CD8^+^ T cell differentiation. In contrast, relatively little is known about the tolerant state, and how it fits into the global differentiation hierarchy of CD8^+^ T cells. Based on gene expression profiling studies, tolerant CD8^+^ T cells clearly exit the naïve state and actively up-regulate genes that are not induced during either early effector differentiation, or lymphopenia-induced proliferation (56, 131). However, there is substantial confusion within the field regarding the difference between CD8^+^ T cell tolerance and other negative regulatory processes, and terms such as exhaustion and anergy are thus often used interchangeably. This confusion partly stems from how these terms are defined. Many use the term “anergy” to describe any cell that responds poorly to TCR restimulation, and by this definition anergic and exhausted cells are both similar. Conversely, if peripheral tolerance is defined as a diminished self-reactive T cell response in autoimmune models, then arguably exhaustion is a form of peripheral tolerance given that exhaustion occurs within self-reactive effector T cells during autoimmunity and can limit the severity of disease (132–135). Furthermore, there are gross phenotypic similarities between tolerance and exhaustion. Both processes are programmed by persistent TCR signaling (129, 136), are maintained by the inhibitory receptor PD-1 (75–80, 137), can be disrupted by IL-2 treatment (84, 138), and show deficiencies in TCR signaling (71, 139). However, if we define these states based on the “gold standard” mouse models in which they were initially described [i.e., transfer tolerance models described in this review, and the chronic LCMV mouse infection model where exhaustion was first described (140, 141)], then there are fundamental differences between peripheral tolerance and exhaustion. In particular, tolerant CD8^+^ T cells typically do not pass through an effector phase or infiltrate tissues, and thus diverge early from functional effector cells (see discussion earlier). In contrast, exhaustion occurs when established effector CD8^+^ T cells are chronically stimulated, and, unlike peripheral tolerance, the first ∼5 days of differentiation are largely indistinguishable between exhausted and fully functional effector CD8^+^ T cells (142–145), although recent evidence does suggest some degree of commitment to the exhausted state by day 5 post-infection in chronic infection models (146). Even after cells have entered an exhausted state, they still retain some capacity to kill targets and produce cytokines, unlike tolerant cells which have much more profound defects in effector function (55, 56). Consistent with these differences, the gene expression profiles of tolerant CD8^+^ T cells is distinct from that of exhausted CD8^+^ T cells found in both tumors and chronic viral infection (56, 147–149). For example, in addition to diminished cytokine/chemokine expression relative to exhausted cells, tolerant cells do not up-regulate certain key inhibitory receptors expressed during exhaustion, such as CD244 (also 2B4), CD160, and CD39 (56), suggesting non-overlapping mechanisms of negative regulation. We would argue that tolerance and exhaustion likely evolved for fundamentally different reasons, so it stands to reason that they are phenotypically distinct. Exhausted cells contribute to immune control of chronic infections and tumors, although their function is tempered to limit immunopathology (5). In contrast, tolerant cells are often actively dangerous to the host, and thus need to be prevented from participating in any immune response. Thus, it is likely important to host survival that tolerant cells have far more limited effector functions than exhausted cells.

Despite their differences, there is overlap in gene expression between tolerance and exhaustion, suggesting that at least some regulatory pathways may be shared between both states (56, 147, 148). Consistent with this, as noted earlier, similar NFAT-dependent regulatory programs appear to operate in both tolerance and exhaustion. NFAT homodimers directly induce expression of the inhibitory receptors PD-1, LAG3, and TIM3 within exhausted cells (98). Furthermore, the NFAT-induced transcription factors EGR2 and NR4A1 that contribute to tolerance induction are also induced in exhaustion and, at least in the case of NR4A1, actively enforce the exhausted phenotype (110, 112, 144). Finally, the transcription factor TOX that was recently identified as a master regulator of exhaustion (145, 150–153) is also induced during CD8^+^ T cell tolerance (56). Nevertheless, it is dangerous to assume that these transcription factors target the same genes in both tolerance and exhaustion as this is yet to be proven. In fact, preliminary evidence suggests that there are substantial differences in the gene programs controlled by these factors in both states. For example, functionally important direct NFAT gene targets during exhaustion, such as TIM3, IRF4, and BATF (98, 154), are minimally induced during tolerance (56). Moreover, we have recently found that although EGR2 controls exhausted CD8^+^ T cell differentiation, it targets a fundamentally different gene program in exhaustion versus anergy (I.A. Parish, unpublished results). Thus, it remains unclear whether there is a meaningful overlap between the gene programs that enforce tolerance and exhaustion.

T cell senescence is another form of T cell negative regulation often discussed in the context of cancer (155), and its relationship to exhaustion, effector differentiation and tolerance is once again unclear. Broadly speaking, senescence refers to cell cycle arrest downstream of either DNA damage, or replication-associated telomere erosion, in a manner typically dependent on DNA response programs (e.g., p53 and p16-dependent pathways) (156). However, cell cycle exit is a normal feature of terminal T cell differentiation down multiple fates, as non-proliferative, terminally differentiated cell subsets are found during both exhaustion and effector differentiation (157–160). Consistent with this, TIM3 and KLRG1, which mark terminally differentiated exhausted and effector cells, respectively, are also regarded as markers of T cell senescence (155). Thus, senescence may normally occur during T cell terminal differentiation regardless of fate, and it is arguably more accurate to consider senescence as a cellular process, like apoptosis, rather than a distinct T cell fate. Nevertheless, the senescence process does not appear to be required for tolerance induction. Within a CD8^+^ T cell deletional tolerance model, p53 loss was unable to disrupt the tolerance process, and had little impact upon cell death (124). Furthermore, unlike in anergy models where there are specific defects in TCR-induced ERK induction (see discussion above), senescent T cells have elevated MAPK/ERK activity that actively enforces the senescent state (161, 162). Finally, gene expression profiling of tolerant CD8^+^ T cells failed to reveal elevated expression of the DNA damage or cell cycle arrest gene signatures commonly seen during senescence (56), and anergic CD8^+^ T cells can re-enter the cell cycle (131). Therefore, existing data suggests that the senescence process is dispensable for tolerance induction.

Overall, more comprehensive phenotyping and functional studies are needed to map the differences between tolerant cells and other differentiation fates. In particular, detailed single cell transcriptomic profiling of tolerance is required to more precisely determine whether there are shared cell sub-populations between tolerance and other fates, and to define specific gene signatures that can selectively identify tolerant cells. This latter point is particularly salient given the large number of scRNAseq studies being conducted; there are currently no “gold standard” gene signatures to definitively identify tolerant CD8^+^ T cell clusters. These studies are important if we are to learn more about peripheral tolerance in the context of tumor immunotherapy as is discussed in the remainder of this review.

## Peripheral Tolerance as an Immune Evasion Mechanism for Tumors

**FIGURE 3:**
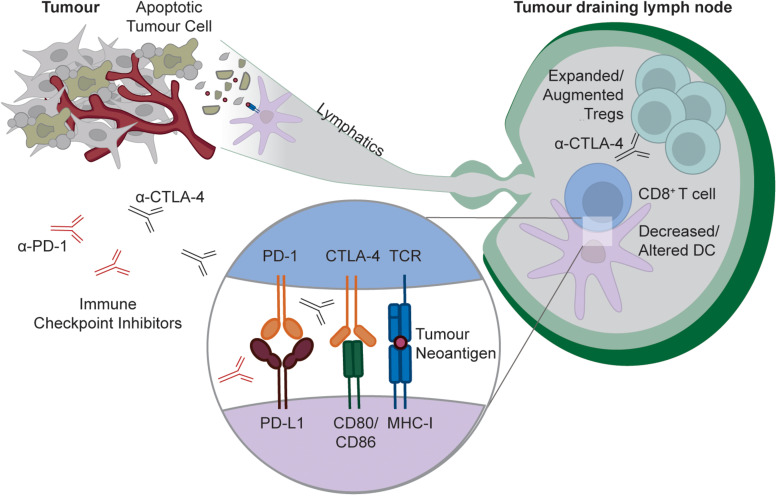
Targeting peripheral tolerance to tumor antigens by checkpoint blockade. As with any self-tissue, tumors can constitutively release cellular debris containing tumor neo-antigens (likely downstream of apoptotic cell death), and these antigens can be captured by DCs for presentation to naïve CD8^+^ T cells within the tumor draining lymph node. In some tumors, tumor antigen presentation is tolerogenic, either because tumors insufficiently activate the innate immune system, or because tumors actively modulate the lymph node environment to limit immunity by decreasing or altering DCs, and/or increasing or augmenting Tregs. Checkpoint blockade with α-PD-1 or α-CTLA4 can disrupt this tolerance process by either interfering with the delivery of negative signals to the T cell by the DC, or, in the case of CTLA-4 blockade, through active Treg targeting and depletion.

For many years, it was thought that tumors are too antigenically similar to “self” tissues to elicit an immune response. However, it is now clear that tumors can be recognized as “foreign” by the immune system, and that evasion of anti-tumor immunity is required for cancer growth (163). Somatic mutations that accumulate during tumor cell transformation generate “foreign” cancer-specific T cell epitopes, and thus tumors with high mutational burdens can provoke antigen-specific T cell responses (164). Furthermore, in some cases, T cell responses even occur against unmutated self-antigens within tumors [e.g., Melan-A/MART1 in melanoma (165–167)]. Initiating, reinvigorating and amplifying T cell responses against tumor antigens is one of the central aims of current cancer immunotherapy approaches. Much of the recent literature has focused on how tumors may hijack exhaustion to evade immune control by tumor infiltrating T cells (5), or how tumors may exclude effector T cells from the tumor (168). In contrast, the degree to which peripheral tolerance to tumor antigens in the draining lymph node contributes to tumor immune evasion is less well defined. Small, non-inflamed and non-necrotic tumors would not be expected to activate the innate immune system, and thus will likely induce peripheral tolerance to any released tumor antigens within the draining lymph node. Additionally, tumors often actively inhibit immune activation, including in the tumor draining lymph node (169–173). This can be due to a range of effects within the draining lymph node (Figure 3), including alteration of DC phenotype or number (174–177), and increased Treg number or function (176–180). These effects appear to be magnified by tumor metastasis to the draining lymph node (176, 181–183), and may be further exacerbated by systemic tumor-mediated immunosuppression of DC function (184).

Consistent with these observations, some tumors are capable of inducing peripheral tolerance. In certain mouse models, tumors constitutively induce peripheral tolerance to tumor-derived antigens within the draining lymph node (173, 185–187). Conversely, though, other tumors constitutively prime anti-tumor effector T cell response in the draining lymph node (188–192). The factors that determine whether or not a tumor primes an immune response are unclear. Ectopic secretion of immunoregulatory factors by some tumors may favor tolerance in the draining lymph node (173). Alternatively, tumor mutational load may be a contributing factor. Adoptively transferring large numbers of naïve CD8^+^ TCR transgenic cells can override peripheral tolerance in certain tumor and self-tolerance models, whereas tolerance remains intact after transfer of more physiologically relevant numbers of T cells (54, 185). In transplantable tumor models, heavily mutated tumors will simultaneously activate more T cells within the draining lymph node than less mutated tumors due to the larger number of “foreign” neo-antigens, and this could override peripheral tolerance by a similar mechanism. This is a potential caveat of transplantable tumor models that is unlikely to be observed in spontaneous tumors, where mutations (and the T cell responses to them) will accrue slowly over time. In support of this idea, in some systems, spontaneous tumors that are not immunogenic *in situ* prime a T cell response once transplanted into a new host (191). This also means that the influence of peripheral tolerance on immune evasion may be underestimated in transplantable tumor models, and that this immune evasion strategy may be much more prevalent in patients than predicted by most commonly used tumor models. Nevertheless, some *in situ*, inducible tumor models prime T cell immunity (190), indicating that further work is needed to rigorously identify the factors that determine whether or not a tumor induces peripheral tolerance.

Lymph node environments that favor tolerance may not only limit initial priming of effector responses, they may also limit established memory CD8^+^ T cell responses. Circulating memory CD8^+^ T cells can be tolerised by tissue-restricted self-antigens (193). Thus, a tumor that is largely cleared by a primary response could in theory tolerise the circulating memory CD8^+^ T cell pool within the draining lymph node if the tumor recurs at a later timepoint. However, the capacity of circulating memory cells to be tolerised varies between models, with another study demonstrating that memory cells ignore DC-expressed self-antigen and fail to be tolerised (194). It is thus unclear to what degree tolerance of memory CD8^+^ T cells contributes to tumor immune evasion, as this process may only occur under very specific circumstances. Moreover, tissue resident memory CD8^+^ T cells from viral models are largely resistant to tolerance induced by cross-presented antigen (195), which is important given that tissue resident memory cells play an important role in tumor control (196). Resistance of tissue resident cells to tolerance induction could be because they remain in the tissue and do not access cross-presenting tolerogenic cDC1s within the draining lymph node. Whether the cross-presenting cDC1s that are found within some tumors (197) can similarly induce tolerance of tissue resident memory cells is unknown. Overall, though, the presence of cross-presenting cDC1s within the tumor is usually associated with a better prognosis (197) arguing that tumor infiltrating cDC1s typically contribute to CD8^+^ T cell priming rather than tolerance. Furthermore, there is no compelling evidence that tolerance induction occurs within tumors. For example, tumor-infiltrating CD8^+^ T cells typically have an exhausted rather than a tolerant gene signature (149). Collectively, these data suggest that peripheral tolerance is restricted to the tumor draining lymph node, and that inhibition of resident memory CD8^+^ T cell function within the tumor is largely due to exhaustion.

Finally, in addition to peripheral deletion and anergy, some tumors evade immunity by exploiting immune ignorance (186, 198). This could be due to a range of factors, including tumor-mediated DC depletion from the draining lymph node (see discussion above), insufficient antigen release to activate neoantigen-specific CD8^+^ T cells that are often low avidity (186), or potentially antigen sequestration within certain organs [e.g., brain (41)]. It is often assumed that antigen release by CTL in these contexts will trigger “epitope spreading” by priming new anti-tumor responses within the draining lymph node, however, antigen release by CTL killing alone is insufficient to activate the innate immune system, and thus leads to tolerance in the draining lymph node (58). Consistent with this idea, a recent study leveraging CAR T cells to recruit more DCs to tumors was only capable of eliciting substantial epitope spreading within the draining lymph node when co-delivered with immune agonists that trigger DC immunogenicity and engage T cell co-stimulatory signaling (199). Thus, tumors can exploit a range of peripheral tolerance mechanisms to evade immunity.

## The Peripheral Tolerance Checkpoint as a Target for Immunotherapy

Given its role in tumor immune evasion, targeting of the peripheral tolerance immune checkpoint may have therapeutic potential. In fact, it is possible that disruption of peripheral tolerance contributes to the efficacy of existing therapeutic approaches. As noted earlier, despite the initial focus on the role of tumor infiltrating CD8^+^ T cells in the response to checkpoint blockade, emerging data suggest that responses initiated outside of the tumor are a major contributor to the efficacy of therapy (11–13). While these effects could be due to therapy-induced expansion of circulating populations of exhausted CD8^+^ T cells, it is also possible that peripheral tolerance disruption in the tumor-draining lymph node contributes to therapy-induced responses. Indeed, as noted earlier, both PD-1 and CTLA-4 are required for peripheral CD8^+^ T cell tolerance, so disrupting these pathways during checkpoint blockade should globally impair peripheral tolerance against both non-tumor and tumor-derived antigens (Figure 3). Arguably the strongest evidence that checkpoint blockade globally disrupts peripheral tolerance is that the primary side-effects of therapy are inflammatory and autoimmune symptoms, including organ-specific CD8^+^ T cell mediated autoimmune diseases such as Vitiligo and Type I Diabetes (200). While factors beyond disruption of CD8^+^ T cell peripheral tolerance could contribute to these phenomena, peripheral tolerance disruption almost certainly at least amplifies these side-effects. Thus, existing cancer immunotherapies likely disrupt peripheral tolerance, although the contribution of tolerance disruption to the efficacy of therapy remains unclear. More compelling evidence demonstrating the induction of *de novo* anti-tumor responses by therapy-induced disruption of peripheral CD8^+^ T cell tolerance to tumor antigens within the draining lymph node is required.

Despite their capacity to disrupt the tolerance process, existing checkpoint blockade approaches still have their limitations. In particular, PD-1 and/or CTLA-4 blockade are only effective at disrupting tolerance when it is initially being induced. Once an anergic population of CD8^+^ T cells is established, cells are generally refractory to re-activation, even after checkpoint blockade (76, 78). Vaccination approaches can resolve this issue, as vaccination with the specific antigen recognized by the anergic population, in combination with checkpoint blockade, was capable of rescuing function and triggering anti-tumor activity within these anergic cell populations (76). This may be due to direct recognition of vaccine-derived antigen by the anergic CD8^+^ T cells, or indirectly due to the induction of antigen-specific CD4^+^ T cell help, which can also override tumor-induced peripheral tolerance and trigger tumor control (185, 186, 189). In future, a better understanding of the molecular pathways that initiate and enforce tolerance could identify additional cancer immunotherapy approaches for more aggressive therapeutic disruption of the peripheral tolerance checkpoint.

## Outstanding Questions and Future Challenges

Based on abundant evidence within the field, we argue that a better understanding of the peripheral tolerance checkpoint may have profound implications for cancer immunotherapy. However, much remains to be learned about the CD8^+^ T cell peripheral tolerance process. Despite circumstantial data hinting at the importance of certain pathways in programming tolerance, there is little or no functional data identifying the transcriptional and molecular master regulators of the tolerant state. Without these data, and a more comprehensive study of the epigenetic landscape and stability of tolerant cells, it is difficult to define how similar or different tolerant cells are from other states of CD8^+^ T cell differentiation, such as exhaustion. In the context of cancer, it remains unclear how prevalent peripheral tolerance is as a cancer immune evasion strategy, and it is unclear what factors determine whether or not a tumor induces tolerance. Furthermore, the exact contribution of therapy-induced peripheral tolerance disruption to the anti-tumor efficacy of checkpoint blockade is also unclear. These studies cannot be rigorously conducted until “gold standard” gene signatures that specifically identify tolerant cells are defined.

Finally, the therapeutic potential of differentially targeting tolerance and exhaustion, or more aggressively disrupting peripheral tolerance, remains unexplored. Pinpointing approaches for differentially targeting these states has potentially profound clinical implications, and should thus be a priority for the field. For example, it may not be necessary to disrupt peripheral tolerance within patient populations that already have evidence of a strong anti-tumor response (i.e., a heavily T cell infiltrated tumor). Selective disruption of exhaustion in this context could preserve the anti-tumor efficacy of therapy, while mitigating autoimmune side-effects. Alternatively, identification of shared regulatory pathways between both states may reveal better drug targets for more aggressive immunotherapy, potentially in combination with vaccination, within patients with limited T cell tumor infiltration and elevated peripheral tolerance within the tumor draining lymph node. This latter group of patients is commonly refractory to existing checkpoint blockade approaches, but a more aggressive approach to disrupting tolerance could overcome these problems. Thus, we conclude that a more refined and targeted approach to immunotherapy aimed at differentially modulating peripheral tolerance has the potential to maximize anti-tumor efficacy while limiting deleterious side-effects.

## Author Contributions

SN, JT, and IP wrote the manuscript. SN and IP prepared the figures. All authors contributed to the article and approved the submitted version.

## Conflict of Interest

The authors declare that the research was conducted in the absence of any commercial or financial relationships that could be construed as a potential conflict of interest.
